# Placental biomarker and fetoplacental Doppler abnormalities are strongly associated with placental pathology in pregnancies with small‐for‐gestational‐age fetus: prospective study

**DOI:** 10.1002/uog.29237

**Published:** 2025-05-07

**Authors:** J. Hong, K. Crawford, E. Cavanagh, V. Clifton, F. da Silva Costa, A. V. Perkins, S. Kumar

**Affiliations:** ^1^ Mater Research Institute University of Queensland Brisbane Queensland Australia; ^2^ School of Medicine The University of Queensland Herston Queensland Australia; ^3^ Department of Obstetrics and Gynecology, Faculty of Medicine University of Malaya Kuala Lumpur Malaysia; ^4^ School of Medicine and Dentistry Griffith University and Maternal Fetal Medicine Unit, Gold Coast University Hospital Gold Coast Queensland Australia; ^5^ School of Health University of the Sunshine Coast Sippy Downs Queensland Australia; ^6^ NHMRC Centre for Research Excellence in Stillbirth, Mater Research Institute University of Queensland Brisbane Queensland Australia

**Keywords:** cerebroplacental ratio, fetal growth restriction, maternal vascular malperfusion, placental dysfunction, placental growth factor, placental pathology, pregnancy, small‐for‐gestational age, soluble fms‐like tyrosine kinase‐1, umbilical artery Doppler

## Abstract

**Objective:**

Placental dysfunction can result in small‐for‐gestational age (SGA) or fetal growth restriction (FGR). The aim of this prospective cohort study was to assess the association of the cerebroplacental ratio (CPR) and other more conventional fetoplacental Doppler indices, circulating placental growth factor (PlGF) levels and soluble fms‐like tyrosine kinase‐1 (sFlt‐1)/PlGF ratio, with specific placental abnormalities in a large cohort of pregnancies with an SGA/FGR fetus.

**Methods:**

This was a prospective cohort study of singleton pregnancies with a SGA/FGR fetus conducted at the Centre for Maternal and Fetal Medicine at the Mater Mother's Hospital, Queensland, Australia. Multivariable logistic regression with adjustment for pre‐eclampsia was used to evaluate the effect of CPR < 5^th^ centile, umbilical artery Doppler abnormality (defined as umbilical artery (UA) pulsatility index (PI) > 95^th^ centile, or absent or reversed end‐diastolic flow), mean uterine artery (UtA) PI > 95^th^ centile and abnormal placental biomarkers (PlGF level < 100 ng/L and sFlt‐1/PlGF ratio > 5.78 if gestational age < 28 weeks or > 38 if gestational age ≥ 28 weeks) on the following placental abnormalities, classified based on the Amsterdam Placental Workshop Group Consensus criteria: placental maternal vascular malperfusion (MVM), fetal vascular malperfusion (FVM), villitis of unknown etiology (VUE), chronic histiocytic intervillositis (CHI) and delayed villous maturation (DVM).

**Results:**

Among the 367 women included in this study, MVM was present in 159 (43.3%) placentae, FVM in 20 (5.4%), VUE in 49 (13.4%), DVM in 19 (5.2%) and CHI in six (1.6%). Compared to SGA controls with normal fetoplacental Doppler and placental biomarkers, CPR < 5^th^ centile (adjusted odds ratio (aOR), 3.17 (95% CI, 1.95–5.16); *P* < 0.001), abnormal UA Doppler (aOR, 2.97 (95% CI, 1.80–4.90); *P* < 0.001) and mean UtA‐PI > 95^th^ centile (aOR, 5.42 (95% CI 2.75–10.70); *P* < 0.001) were associated with higher odds of placental abnormality. The odds of MVM specifically were significantly higher when CPR < 5^th^ centile (aOR, 2.47 (95% CI, 1.64–4.33); *P* < 0.001), abnormal UA Doppler (aOR, 3.13 (95% CI, 1.91–5.12); *P* < 0.001) or mean UtA‐PI > 95^th^ centile (aOR, 4.01 (95% CI, 2.25–7.13); *P* < 0.001) was present. The odds of placental abnormality were also significantly higher if PlGF levels were < 100 ng/L (aOR, 3.66 (95% CI, 2.22–6.06); *P* < 0.001) or the sFlt‐1/PlGF ratio was elevated (aOR, 3.74 (95% CI, 2.17–6.43); *P* < 0.001). The odds of MVM were also higher in women with PlGF < 100 ng/L (aOR, 2.89 (95% CI, 1.72–4.85); *P* < 0.001) and elevated sFlt‐1/PlGF ratio (aOR, 3.15 (95% CI, 1.83–5.45); *P* < 0.001).

**Conclusion:**

In pregnancies with SGA/FGR fetus, mean UtA‐PI > 95^th^ centile, abnormal UA Doppler, CPR < 5^th^ centile, PlGF < 100 ng/L and elevated sFlt‐1/PlGF ratio were all strongly associated with placental abnormality, particularly MVM. © 2025 The Author(s). *Ultrasound in Obstetrics & Gynecology* published by John Wiley & Sons Ltd on behalf of International Society of Ultrasound in Obstetrics and Gynecology.

## INTRODUCTION

Both small‐for‐gestational age (SGA) and fetal growth restriction (FGR) are major determinants of adverse perinatal outcome^1^, ^2^. Some SGA fetuses, however, may not be growth‐restricted and can have relatively normal intrauterine growth velocity and pregnancy outcome^3^, ^4^. Conversely, some appropriate‐for‐gestational‐age (AGA) fetuses could have unrecognized suboptimal intrauterine growth, placing them at increased risk of morbidity and mortality despite having a birth weight above the 10^th^ centile. Given the frequent finding of cerebral redistribution in fetuses that are compromised *in utero*, a low cerebroplacental ratio (CPR) (the ratio of the fetal middle cerebral artery (MCA) pulsatility index (PI) to the umbilical artery (UA) PI) is now assumed to be a reliable marker of suboptimal intrauterine growth secondary to placental dysfunction, even in fetuses that are not SGA^5^, ^6^, ^7^, ^8^, ^9^. However, despite this presumption, there is a paucity of data confirming an association between a low CPR and the pathognomonic macro‐ and microscopic abnormalities typically seen in FGR placentae. A low CPR is associated with perinatal mortality, intrapartum fetal compromise and adverse neonatal outcome in both SGA and AGA fetuses^5^, ^10^, ^11^, ^12^.

Defective implantation and invasion of cytotrophoblasts into the myometrium and subsequent abnormal spiral artery remodeling lead to placental dysfunction^13^, ^14^. Disruption in placental vasculogenesis and angiogenesis followed by placental ischemia result in low levels of placental growth factor (PlGF) and elevated levels of its antiangiogenic counterpart, soluble fms‐like tyrosine kinase‐1 (sFlt‐1)^15^, ^16^. In the fetus, the severity of placental dysfunction is often reflected by raised UA‐PI and uterine artery (UtA) PI^17^, ^18^, ^19^, ^20^, which are associated with typical placental abnormalities^21^, ^22^, ^23^.

Typical abnormalities seen in FGR placentae include maternal vascular malperfusion (MVM), fetal vascular malperfusion (FVM), villitis of unknown etiology (VUE), chronic histiocytic intervillositis (CHI) and delayed villous maturation (DVM)^21^, ^23^, ^24^, ^25^, ^26^. Although several studies^5^, ^12^, ^27^, ^28^, ^29^, ^30^ have demonstrated an association between low CPR and adverse pregnancy outcome, only a few^31^, ^32^ have investigated the association between low CPR and specific placental abnormalities. The aim of this study was to assess the association of CPR and other fetoplacental Doppler indices, circulating PlGF levels and sFlt‐1/PlGF ratio with placental abnormalities, in a large cohort of well‐characterized SGA/FGR pregnancies.

## METHODS

### Study design

This observational, prospective cohort study was conducted at the Centre for Maternal and Fetal Medicine at the Mater Mother's Hospital in Queensland, Australia, from May 2022 to March 2024. Women attending a dedicated fetal growth clinic with a singleton pregnancy without known genetic syndrome, aneuploidy, major structural malformation or fetal infection were eligible to participate. Women without a placental histopathological report were excluded.

SGA was defined as abdominal circumference (AC) or estimated fetal weight (EFW) < 10^th^ centile. FGR was defined according to the Delphi consensus of Gordijn *et al*.^33^ as follows. Early FGR (diagnosed < 32 + 0 weeks) was defined as: (1) AC or EFW < 3^rd^ centile; (2) absent end‐diastolic flow in the UA; or (3) AC or EFW < 10^th^ centile in combination with UtA‐PI > 95^th^ centile and/or UA‐PI > 95^th^ centile. Late FGR (diagnosed ≥ 32 + 0 weeks) was defined as: (1) AC or EFW < 3^rd^ centile; or (2) at least two of the following: AC or EFW < 10^th^ centile; AC or EFW crossing more than two growth quartiles; and CPR < 5^th^ centile or UA‐PI > 95^th^ centile. Gestational age was calculated based on first‐trimester crown–rump length measurement. Ethical and governance approval were obtained from the Mater Misericordiae Limited Human Research Ethics Committee (HREC/MML/66263 (V3)) and the Mater Governance Office, respectively. All women provided written informed consent to participate.

At recruitment, all women had fetal biometry^34^ (biparietal diameter, head circumference, AC, femur length) measured and EFW was calculated using Hadlock's formula^35^. Fetal growth assessment was performed every 2–4 weeks depending on the severity of the fetal condition. In addition, the deepest pocket of amniotic fluid, UtA‐PI, UA‐PI and MCA‐PI were measured, CPR was calculated^36^, ^37^ and the presence of absent or reversed end‐diastolic flow (AREDF) in the UA was recorded. All ultrasound assessments were performed using a Voluson™ E10 (GE Healthcare, Zipf, Austria) ultrasound platform and ultrasound data were stored in a dedicated ultrasound reporting system (Viewpoint®; GE HealthCare).

Maternal blood samples for PlGF and sFlt‐1 level assessment were collected in vacutainer EDTA tubes (Becton Dickinson Labware, Franklin Lakes, NJ, USA) and measured using the B·R·A·H·M·S KRYPTOR PLUS system (Thermo Fisher Scientific, BRAHMS GmbH, Hennigsdorf, Germany). Placental biomarkers were measured at the first visit and then repeated every 4 weeks. Maternal serology for evidence of toxoplasmosis, rubella, cytomegalovirus, herpes simplex and syphilis infection was also performed.

Ultrasound findings were reviewed by maternal–fetal medicine specialists who were blinded to placental biomarker results, and all clinical decisions, including intensity of antenatal surveillance and timing of birth, were based on fetal ultrasound indices and/or the overall maternal clinical condition, based on the International Society of Ultrasound in Obstetrics and Gynecology (ISUOG) practice guidelines.^38^ If delivery between 24 + 0 and 34 + 6 weeks of gestation was anticipated, a course of antenatal corticosteroids (two doses of 12‐mg intramuscular betamethasone, 24 h apart) was administered^39^. Magnesium sulfate prophylaxis for fetal neuroprotection (intravenous infusion of 4‐g bolus over 20 min, followed by 1 g/h for up to 24 h) was also given if delivery < 30 weeks was required^40^.

Placentae with an attached umbilical cord were collected immediately after birth and transported fresh to the pathology laboratory for histopathological assessment. All placental histopathological examinations were performed by a perinatal pathologist, who was blinded to the ultrasound findings, using a standardized protocol for classification of placental abnormalities based on the Amsterdam Placental Workshop Group Consensus Criteria^41^. The placenta, umbilical cord and fetal membranes were examined for gross morphological lesions. Subsequently, the placental disc was sectioned into 8‐mm slices for further histological analysis of parenchymal lesions. Three samples of normal parenchymal blocks (two sections from the central disc and one from the cord insertion site) and any identified lesions, a membrane roll and two sections of umbilical cord were obtained (one from the fetal end and another 5 cm from the cord insertion site). Trimmed placental‐weight centiles were calculated using appropriate gestational‐age charts^42^. The study participants' demographic and pregnancy information as well as pregnancy outcome were recorded in an electronic form using the REDCap™ platform (https://redcap.health.uq.edu.au/). Sex‐specific Australian birth‐weight population charts were used^43^.

### Exposures and confounders

Fetoplacental Doppler exposures were CPR, UA‐PI and UtA‐PI *Z*‐scores, CPR < 5^th^ centile, abnormal UA Doppler (UA‐PI > 95^th^ centile or AREDF) and mean UtA‐PI > 95^th^ centile. Placental biomarker exposures were PlGF level and sFlt‐1/PlGF ratio *Z‐*scores, PlGF < 100 ng/L^18^, ^44^, ^45^, ^46^ and sFlt‐1/PlGF ratio > 5.78 for gestational age < 28 weeks and > 38 for gestational age ≥ 28 weeks^16^, ^18^, ^45^, ^46^. Abnormal Doppler thresholds were defined as CPR < 5^th^ centile, UA‐PI > 95^th^ centile, AREDF and mean UtA‐PI > 95^th^ centile^33^, ^38^, ^47^. Abnormal placental biomarker cut‐offs were defined as PlGF level < 100 ng/L^18^, ^44^, ^45^, ^46^ and elevated sFlt‐1/PlGF ratio (> 5.78 for gestational age < 28 weeks and > 38 for gestational age ≥ 28 weeks); these have been used previously and validated in observational studies defining biomarker gestational‐age screening thresholds for FGR (with or without pre‐eclampsia)^16^, ^18^, ^45^, ^46^. Pre‐eclampsia was the only clinically relevant confounder^48^.

### Outcomes

The primary outcomes were (1) any type of placental abnormality^41^, ^49^ (MVM, FVM, VUE, DVM and/or CHI, i.e. in isolation or in combination) and (2) MVM specifically. If multiple abnormalities were present in the same placenta, primary categorization was based on the severity, extent and grading of the predominant placental lesion^23^. MVM was characterized by placental hypoplasia, infarction, retroplacental hemorrhage, distal villous hypoplasia, accelerated villous maturation, syncytial knots and/or decidual arteriopathy. FVM was characterized by arterial or venous thrombosis, avascular villi, intramural fibrin deposition, villous stromal‐vascular karyorrhexis, stem vessel obliteration and/or vascular ectasia. DVM was defined as monotonous villous population (minimum of 10 villi) when present in at least 30% of a full‐thickness parenchymal slide. VUE was defined as inflammation affecting contiguous villi (usually lymphohistiocytic) in the absence of clinical and histopathological signs of infection. CHI was defined as maternal histiocytic (CD68+) infiltrate occupying at least 5% of the intervillous space in the absence of clinical and histopathological signs of infection.

### Statistical analysis

Maternal demographic variables and pregnancy outcomes stratified by fetoplacental Doppler parameters and placental biomarkers are presented as *n* (%), mean ± SD or median (interquartile range), according to their distribution. Pregnancy outcomes were compared by exposure status using univariable logistic regression analysis. The incidence (rate per 100) of placental abnormalities (MVM, FVM, VUE, DVM and CHI), stratified by normal/abnormal cut‐off values of fetoplacental Doppler parameters and placental biomarkers, is presented in bar graphs. The unadjusted univariable association between CPR, UA‐PI, mean UtA‐PI, PlGF and the sFlt‐1/PlGF ratio at the final assessment before birth was assessed for placental abnormality and for MVM using logistic regression, accounting for clustering at the patient level. Multivariable logistic regression models were built, adjusting for pre‐eclampsia on the basis that it is a clinically relevant confounder^48^. Appropriate specification of models was determined using the Hosmer and Lemeshow goodness‐of‐fit test^50^, with acceptability set at *P* > 0.05. Boxplots of raw values of fetoplacental Doppler parameters and placental biomarkers at the final assessment before birth are presented for placental abnormality and for MVM. All data were analyzed using Stata 18® (Statacorp LLC, College Station, TX, USA). The STROBE (Strengthening the Reporting of Observational Studies in Epidemiology) statement was followed for reporting of the study results^51^.

## RESULTS

**Figure 1:**
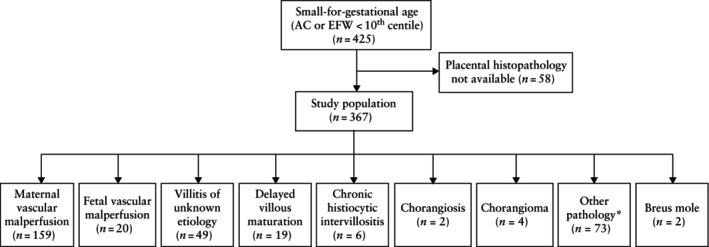
Flowchart summarizing study population and placental pathology. Thirty‐three placentae were without identifiable predominant pathology and therefore could not be classified. *Other pathology included ascending intrauterine infection, subchorionic or intraparenchymal hemorrhage, and villous hydrops of uncertain significance. AC, abdominal circumference; EFW, estimated fetal weight.

In total, 367 women with a singleton SGA pregnancy and available placental histopathological information were included in the study. Table 1 presents the demographic characteristics of the study population, stratified by fetoplacental Doppler findings and placental biomarkers. Overall, 148 (40.3%) pregnancies had CPR < 5^th^ centile, 139 (37.9%) had abnormal UA Doppler, 94 (25.6%) had mean UtA‐PI > 95^th^ centile, 181 (49.3%) had a PlGF level < 100 ng/L and 141 (38.4%) had an elevated sFlt‐1/PlGF ratio. MVM was present in 159 (43.3%) cases, FVM in 20 (5.4%), VUE in 49 (13.4%), DVM in 19 (5.2%) and CHI in six (1.6%). In 81 (22.1%) placentae, other abnormality (chorangiosis, choriangioma, Breus mole, ascending intrauterine infection, subchorionic or intraparenchymal hemorrhage, or villous hydrops of uncertain significance) was present (Figure 1).

**Table 1:** Characteristics of study population of pregnancies with small‐for‐gestational‐age (SGA) fetus stratified by fetoplacental Doppler parameters and placental biomarkers

Characteristic	Total (*n* = 367)	CPR < 5^th^ centile (*n* = 148)	Abnormal UA Doppler* (*n* = 139)	Mean UtA‐PI > 95^th^ centile (*n* = 94)	PlGF < 100 ng/L (*n* = 181)	High sFlt‐1/PlGF ratio† (*n* = 141)
Maternal age (years)	30.8 ± 6.2	31.3 ± 6.2	32.3 ± 6.1	31.7 ± 6.1	31.2 ± 6.7	31.0 ± 6.7
Nulliparity	198 (54.0)	84 (56.8)	79 (56.8)	48 (51.1)	109 (60.2)	84 (59.6)
GA at recruitment (weeks)	32.0 (28.0–35.0)	31.0 (28.0–34.0)	30.0 (27.0–33.0)	30.0 (26.0–33.0)	30.0 (26.0–34.0)	30.0 (26.0–34.0)
BMI at recruitment (kg/m^2^)	25.8 (21.7–30.9)	27.0 (22.5–32.0)	27.3 (23.5–32.4)	29.0 (24.1–34.2)	27.6 (23.2–32.8)	26.8 (22.4–32.4)
MAP at recruitment (mmHg)	83.3 (77.0–93.0)	90.0 (82.0–97.2)	89.0 (80.0–97.0)	92.0 (85.0–102.0)	89.2 (81.0–97.0)	89.3 (82.0–97.0)
Born in Australia						
Non‐indigenous	144 (39.2)	68 (45.9)	59 (42.4)	42 (44.7)	77 (42.5)	59 (41.8)
Indigenous	25 (6.8)	12 (8.1)	9 (6.5)	5 (5.3)	12 (6.6)	11 (7.8)
Born outside Australia						
Asia	124 (33.8)	38 (25.7)	39 (28.1)	23 (24.5)	50 (27.6)	38 (27.0)
Africa	27 (7.4)	7 (4.7)	10 (7.2)	8 (8.5)	14 (7.7)	11 (7.8)
Europe	12 (3.3)	4 (2.7)	6 (4.3)	1 (1.1)	5 (2.8)	2 (1.4)
New Zealand/Pacific/Oceania	21 (5.7)	12 (8.1)	7 (5.0)	9 (9.6)	13 (7.2)	12 (8.5)
North/South America	8 (2.2)	3 (2.0)	5 (3.6)	2 (2.1)	6 (3.3)	5 (3.5)
Middle East	3 (0.8)	3 (2.0)	3 (2.2)	2 (2.1)	2 (1.1)	1 (0.7)
Other	3 (0.8)	1 (0.7)	1 (0.7)	2 (2.1)	2 (1.1)	2 (1.4)
Conception by ART	27 (7.4)	17 (11.5)	18 (12.9)	11 (11.7)	16 (8.8)	8 (5.7)
Previous stillbirth	14 (3.8)	8 (5.4)	9 (6.5)	9 (9.6)	10 (5.5)	10 (7.1)
Previous preterm birth	34 (9.3)	14 (9.5)	14 (10.1)	18 (19.1)	18 (9.9)	16 (11.3)
Previous SGA infant	94 (25.6)	41 (27.7)	38 (27.3)	31 (33.0)	43 (23.8)	34 (24.1)
Hypertension	98 (26.7)	66 (44.6)	61 (43.9)	55 (58.5)	81 (44.8)	60 (42.6)
Pre‐eclampsia	97 (26.4)	68 (45.9)	65 (46.8)	53 (56.4)	79 (43.6)	58 (41.1)
Aspirin use	79 (21.5)	44 (29.7)	49 (35.3)	37 (39.4)	53 (29.3)	39 (27.7)
LMWH use	21 (5.7)	12 (8.1)	14 (10.1)	13 (13.8)	14 (7.7)	9 (6.4)
Diabetes mellitus	99 (27.0)	45 (30.4)	46 (33.1)	28 (29.8)	57 (31.5)	42 (29.8)
Connective tissue disease	10 (2.7)	5 (3.4)	7 (5.0)	7 (7.4)	7 (3.9)	5 (3.5)
Renal disease	8 (2.2)	3 (2.0)	4 (2.9)	3 (3.2)	6 (3.3)	4 (2.8)
Smoker	65 (17.7)	37 (25.0)	26 (18.7)	16 (17.0)	33 (18.2)	28 (19.9)
Alcohol use	8 (2.2)	4 (2.7)	3 (2.2)	2 (2.1)	5 (2.8)	6 (4.3)
Type of FGR‡						
Early‐onset (< 32 weeks)	160 (43.6)	89 (60.1)	93 (66.9)	63 (67.0)	102 (56.4)	77 (54.6)
Late‐onset (≥ 32 weeks)	139 (37.9)	57/148 (38.5)	41 (29.5)	28 (29.8)	63 (34.8)	52 (36.9)
PlGF <100ng/L at recruitment	135/336 (40.2)	93/137 (67.9)	87/130 (66.9)	62/85 (72.9)	—	99/133 (74.4)
PlGF <100ng/L before delivery	181/354 (51.1)	112/142 (78.9)	104/136 (76.5)	75/90 (83.3)	—	135 (95.7)
High sFlt‐1/PlGF ratio† at recruitment	133/335 (39.7)	91/137 (66.4)	84/130 (64.6)	63/84 (75.0)	127/172 (73.8)	—
High sFlt‐1/PlGF ratio† before delivery	141/302 (46.7)	87/119 (73.1)	80/117 (68.4)	56/71 (78.9)	135/155 (87.1)	—
Mean UtA‐PI > 95^th^ centile at recruitment	94 (25.6)	61 (41.2)	60 (43.2)	—	75 (41.4)	56 (39.7)
Mean UtA‐PI > 95^th^ centile before delivery	94 (25.6)	61 (41.2)	57 (41.0)	—	75 (41.4)	56 (39.7)
UA‐PI > 95^th^ centile at recruitment	136 (37.1)	100 (67.6)	—	58 (61.7)	101 (55.8)	77 (54.6)
UA‐PI > 95^th^ centile before delivery	139 (37.9)	112 (75.7)	—	57 (60.6)	104 (57.5)	80 (56.7)
CPR < 5^th^ centile at recruitment	109 (29.7)	—	88 (63.3)	53 (56.4)	85 (47.0)	63 (44.7)
CPR < 5^th^ centile before delivery	148 (40.3)	—	112 (80.6)	61 (64.9)	112 (61.9)	87 (61.7)
PlGF level (ng/L) at recruitment	136.15 (51.25–343.50)	58.60 (27.80–127.10)	56.15 (26.10–143.90)	42.60 (21.30–107.40)	53.20 (27.50–96.10)	54.40 (28.20–102.40)
PlGF level (ng/L) before delivery	93.10 (42.20–260.00)	47.90 (28.10–80.40)	46.55 (26.00–83.25)	37.35 (21.60–72.20)	42.90 (27.20–63.50)	45.10 (28.20–62.60)
sFlt‐1/PlGF ratio at recruitment	14.60 (3.70–92.20)	67.20 (13.70–204.80)	82.15 (10.70–262.40)	132.75 (25.10–373.50)	85.95 (23.05–259.15)	83.30 (25.40–191.20)
sFlt‐1/PlGF ratio before delivery	32.80 (5.80–126.50)	110.35 (37.10–254.00)	110.35 (28.65–310.65)	177.50 (53.30–454.70)	122.55 (61.05–312.85)	122.90 (70.90–251.40)
Mean UtA‐PI at recruitment	0.87 (0.67–1.30)	1.13 (0.82–1.63)	1.16 (0.83–1.67)	1.62 (1.38–1.94)	1.16 (0.80–1.64)	1.15 (0.82–1.59)
Mean UtA‐PI before delivery	0.83 (0.65–1.23)	1.05 (0.76–1.61)	1.08 (0.79–1.62)	1.62 (1.40–1.84)	1.02 (0.75–1.62)	1.01 (0.75–1.50)
UA‐PI at recruitment	1.07 (0.93–1.27)	1.31 (1.11–1.56)	1.34 (1.15–1.60)	1.25 (1.02–1.57)	1.20 (0.98–1.53)	1.19 (0.99–1.45)
UA‐PI before delivery	1.00 (0.85–1.23)	1.30 (1.11–1.62)	1.36 (1.20–1.63)	1.26 (1.01–1.63)	1.17 (0.95–1.58)	1.15 (0.95–1.58)
CPR at recruitment	1.68 (1.28–2.00)	1.19 (0.92–1.50)	1.21 (0.90–1.48)	1.19 (0.87–1.72)	1.37 (1.01–1.82)	1.43 (1.06–1.83)
CPR before delivery	1.51 (1.07–1.90)	1.00 (0.78–1.16)	1.00 (0.77–1.25)	1.08 (0.79–1.50)	1.15 (0.82–1.58)	1.16 (0.82–1.58)

Data are presented as mean ± SD or median (interquartile range) for continuous measures and *n* (%) or *n*/*N* (%) for categorical measures.

* Abnormal umbilical artery (UA) Doppler included UA pulsatility index (PI) > 95^th^ centile or absent or reversed end‐diastolic flow.

† Soluble fms‐like tyrosine kinase‐1 (sFlt‐1)/placental growth factor (PlGF) ratio > 5.78 if gestational age (GA) < 28 weeks or > 38 if GA ≥ 28 weeks.

‡ Based on Delphi consensus criteria.

ART, assisted reproductive technique; BMI, body mass index; CPR, cerebroplacental ratio; FGR, fetal growth restriction; LMWH, low molecular weight heparin; MAP, mean arterial pressure; PTB, preterm birth; SGA, small‐for‐gestational age; UtA, uterine artery.

Rates of previous stillbirth, previous SGA infant and previous preterm birth (PTB) were higher in women with mean UtA‐PI > 95^th^ centile compared to other abnormal fetoplacental Doppler parameters and placental biomarkers (Table 1). Higher rates of hypertension and pre‐eclampsia were seen in women with CPR < 5^th^ centile, abnormal UA Doppler, PlGF < 100 ng/L, elevated sFlt‐1/PlGF ratio and mean UtA‐PI > 95^th^ centile compared to women without the corresponding abnormal marker, with the highest rates seen in those with mean UtA‐PI > 95^th^ centile. Rates of abnormal fetoplacental Doppler parameters and placental biomarkers were higher in early FGR compared with late‐onset disease. There were no cases of toxoplasmosis, rubella, cytomegalovirus, herpes simplex or syphilis infection.

**Figure 2:**
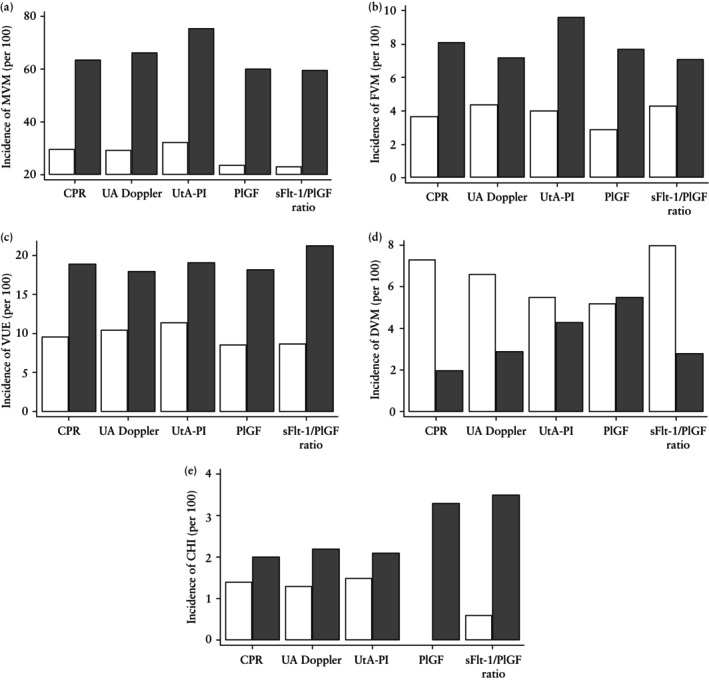
Incidence (per 100) of placental abnormality in pregnancies with small‐for‐gestational‐age fetus, stratified by normality (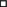) or abnormality (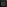) of fetoplacental Doppler parameters (cerebroplacental ratio (CPR), umbilical artery (UA) Doppler and mean uterine artery (UtA) pulsatility index (PI)) and placental biomarkers (placental growth factor (PlGF) and soluble fms‐like tyrosine kinase‐1 (sFlt‐1)/PlGF ratio). Plots show difference in incidence of: (a) maternal vascular malperfusion (MVM), (b) fetal vascular malperfusion (FVM), (c) villitis of unknown etiology (VUE), (d) delayed villous maturation (DVM) and (e) chronic histiocytic intervillositis (CHI). Abnormality of Doppler parameters defined as CPR < 5^th^ centile, abnormal UA Doppler (UA‐PI > 95^th^ centile or absent or reversed end‐diastolic flow) and mean UtA‐PI > 95^th^ centile. Abnormality of placental biomarkers defined as PlGF level < 100 ng/L and sFlt‐1/PlGF ratio > 5.78 for gestational age < 28 weeks and > 38 for gestational age ≥ 28 weeks.

Placental abnormalities stratified by fetoplacental Doppler parameters and placental biomarkers are given in Table 2. MVM lesions were significantly more frequent in cases with CPR < 5^th^ centile *vs* ≥ 5^th^ centile (63.5% *vs* 29.7%; *P* < 0.001), abnormal UA Doppler *vs* normal UA Doppler (66.2% *vs* 29.4%; *P* < 0.001), mean UtA‐PI > 95^th^ centile *vs* ≤ 95^th^ centile (75.5% *vs* 32.2%; *P* < 0.001), PlGF < 100 ng/L *vs* ≥ 100 ng/mL (60.2% *vs* 26.9%; *P* < 0.001) and high *vs* low sFlt‐1/PlGF ratio (59.6% *vs* 33.2%; *P* < 0.001). All cases of CHI had low PlGF. There was a higher incidence of placental MVM, FVM, VUE and CHI in cases with abnormal fetoplacental Doppler parameters or placental biomarkers compared to corresponding controls (i.e. compared to cases with normal corresponding Doppler or biomarker values): the incidence was approximately three times higher for MVM and two times higher for FVM, VUE and CHI if fetoplacental Doppler findings or placental biomarkers were abnormal, compared with corresponding controls (Figure 2). Women with abnormal fetoplacental Doppler findings also had a lower median placental weight compared with controls (CPR < 5^th^ centile *vs* ≥ 5^th^ centile: 272 (IQR, 198–330) g *vs* 363 (IQR, 315–416) g, *P* < 0.001; abnormal UA Doppler *vs* normal UA Doppler: 262 (IQR, 184–317) g *vs* 360 (IQR, 316–410) g, *P* < 0.001; and UtA‐PI > 95^th^ centile *vs* UtA‐PI ≤ 95^th^ centile: 258 (IQR, 177–330) g *vs* 346 (IQR, 298–405) g, *P* < 0.001). Similarly, women with abnormal placental biomarkers had a lower median placental weight compared with controls (PlGF < 100 ng/L *vs* PlGF ≥ 100 ng/L: 280 (IQR, 201–355) g *vs* 370 (IQR, 319–418) g, *P* < 0.001; elevated sFlt‐1/PlGF ratio *vs* normal sFlt‐1/PlGF ratio: 284 (IQR, 204–359) g *vs* 362 (IQR, 314–408) g, *P* < 0.001). Rates of severe (diffuse) MVM abnormalities were higher in cases with abnormal fetoplacental Doppler parameters or placental biomarkers compared to controls without the corresponding abnormal parameter. However, there was no difference in rates of high‐grade (severe) FVM, VUE, DVM or CHI in cases with abnormal fetoplacental Doppler parameters or placental biomarkers compared to controls with normal corresponding parameters, possibly due to the small numbers of patients with severe grades of these abnormalities (Table 2). More granular detail of the types of placental abnormality ais given in Table S1.

**Table 2:** Comparison of placental pathological features in pregnancies with small‐for‐gestational‐age fetus, stratified by fetoplacental Doppler parameters and placental biomarkers

Placental feature	Total (*n* = 367)	CPR < 5^th^ centile (*n* = 148)	*P* *	Abnormal UA Doppler† (*n* = 139)	*P* *	Mean UtA‐PI > 95^th^ centile (*n* = 94)	*P* *	PlGF < 100 ng/L (*n* = 181)	*P* *	High sFlt‐1/PlGF ratio† (*n* = 141)	*P* *
Placental weight (g)	332 (268–386)	272 (198–330)	< 0.001	262 (184–317)	< 0.001	258 (177–330)	< 0.001	280 (201–355)	< 0.001	284 (204–359)	< 0.001
Placental‐weight centile											
< 3^rd^	303 (82.6)	135 (91.2)	< 0.001	124 (89.2)	< 0.001	89 (94.7)	< 0.001	156 (86.2)	< 0.001	122 (86.5)	< 0.001
3^rd^ to < 10^th^	48 (13.1)	10 (6.8)	0.003	14 (10.1)	0.125	2 (2.1)	0.002	17 (9.4)	0.029	12 (8.5)	0.051
10^th^ to < 25^th^	11 (3.0)	3 (2.0)	0.268	1 (0.7)	0.067	1 (1.1)	0.178	5 (2.8)	0.597	4 (2.8)	0.588
Placental thickness (mm)	20 (15–25)	18 (15–24)	< 0.001	17 (14–20)	< 0.001	20 (15–23)	0.089	20 (15–24)	0.013	20 (15–24)	0.077
Cord insertion site											
Marginal	63 (17.2)	30 (20.3)	0.197	27 (19.4)	0.368	19 (20.2)	0.373	32 (17.7)	0.989	24 (17.0)	0.892
Velamentous	5 (1.4)	2 (1.4)	0.956	2 (1.4)	0.883	1 (1.1)	0.808	4 (2.2)	0.227	4 (2.8)	0.174
Cord coiling											
Hypocoiled	32 (8.7)	11 (7.4)	0.368	11 (7.9)	0.549	7 (7.4)	0.506	14 (7.7)	0.399	9 (6.4)	0.406
Hypercoiled	53 (14.4)	17 (11.5)	0.152	16 (11.5)	0.187	10 (10.6)	0.198	28 (15.5)	0.774	21 (14.9)	0.570
True knots	10 (2.7)	3 (2.0)	0.500	3 (2.2)	0.601	2 (2.1)	0.678	6 (3.3)	0.578	6 (4.3)	0.240
Single umbilical artery	10 (2.7)	3 (2.0)	0.500	4 (2.9)	0.894	2 (2.1)	0.678	4 (2.2)	0.679	3 (2.1)	0.594
Maternal vascular malperfusion	159 (43.3)	94 (63.5)	< 0.001	92 (66.2)	< 0.001	71 (75.5)	< 0.001	109 (60.2)	< 0.001	84 (59.6)	< 0.001
Mild (focal)	74 (46.5)	29 (30.9)		31 (33.7)		22 (31.0)		37 (33.9)		34 (40.5)	
Severe (diffuse)	85 (53.5)	65 (69.1)	< 0.001	61 (66.3)	< 0.001	49 (69.0)	< 0.001	72 (66.1)	< 0.001	50 (59.5)	< 0.001
Fetal vascular malperfusion	20 (5.4)	12 (8.1)	0.072	10 (7.2)	0.256	9 (9.6)	0.048	14 (7.7)	0.052	10 (7.1)	0.307
Low (segmental)	16 (80.0)	11 (91.7)		8 (80.0)		7 (77.8)		11 (78.6)		8 (80.0)	
High (global)	4 (20.0)	1 (8.3)	0.149	2 (20.0)	1.000	2 (22.2)	0.827	3 (21.4)	0.948	2 (20.0)	0.692
Villitis of unknown etiology	49 (13.4)	28 (18.9)	0.011	25 (18.0)	0.044	18 (19.1)	0.058	33 (18.2)	0.010	30 (21.3)	0.003
Low	22 (44.9)	11 (39.3)		11 (44.0)		8 (44.4)		12 (36.4)		12 (40.0)	
High	27 (55.1)	17 (60.7)	0.368	14 (56.0)	0.898	10 (55.6)	0.962	21 (63.6)	0.135	18 (60.0)	0.296
Delayed villous maturation	19 (5.2)	3 (2.0)	0.036	4 (2.9)	0.132	4 (4.3)	0.641	10 (5.5)	0.893	4 (2.8)	0.060
Focal	10 (52.6)	2 (66.7)		3 (75.0)		3 (75.0)		7 (70.0)		4 (100.0)	
Diffuse	9 (47.4)	1 (33.3)	0.610	1 (25.0)	0.343	1 (25.0)	0.343	3 (30.0)	0.129	0 (0)	—
Chronic histiocytic intervillositis	6 (1.6)	3 (2.0)	0.629	3 (2.2)	0.542	2 (2.1)	0.665	6 (3.3)	—	5 (3.5)	0.108
Chorangiosis	2 (0.5)	0 (0)	—	0 (0)	—	0 (0)	—	2 (1.1)	0.103	2 (1.4)	0.108
Chorangioma	4 (1.1)	1 (0.7)	0.032	1 (0.7)	0.031	1 (1.1)	0.304	3 (1.7)	0.100	3 (2.1)	0.340
Other pathology‡	73 (19.9)	20 (13.5)	0.013	20 (14.4)	0.041	14 (14.9)	0.162	36 (19.9)	0.830	31 (22.0)	0.753
Breus mole	2 (0.5)	0 (0)	—	0 (0)	—	0 (0)	—	2 (1.1)	0.100	1 (0.7)	0.925

Data are presented as median (interquartile range) for continuous measures and *n* (%) for categorical measures.

*P*‐value generated by logistic regression; *P* < 0.05 considered statistically significant.

* Comparison with control group: cerebroplacental ratio (CPR) ≥ 5^th^ centile for gestational age (GA), umbilical artery (UA) pulsatility index (PI) ≤ 95^th^ centile, mean uterine artery (UtA) PI ≤ 95^th^ centile, placental growth factor (PlGF) ≥ 100 ng/L and soluble fms‐like tyrosine kinase‐1 (sFlt‐1)/PlGF ratio ≤ 5.78 if GA < 28 weeks or ≤ 38 if GA ≥ 28 weeks.

† Abnormal UA Doppler included UA‐PI > 95^th^ centile or absent or reversed end‐diastolic flow.

†Soluble fms‐like tyrosine kinase‐1 (sFlt‐1)/PlGF ratio > 5.78 if GA < 28 weeks or > 38 if GA ≥ 28 weeks.

‡ Ascending intrauterine infection, subchorionic or intraparenchymal hemorrhage, or villous hydrops of uncertain significance.

CPR, cerebroplacental ratio; UtA, uterine artery.

Raw values of fetoplacental Doppler parameters and placental biomarkers at the last measurement before birth are summarized according to presence of placental abnormality and presence of MVM in Figure S1. The adjusted odds ratio (aOR) for pre‐eclampsia associated with a unit increase in the *Z*‐scores and cut‐off points of fetoplacental Doppler parameters and placental biomarkers for the outcomes of placental abnormality and MVM are presented in Table 3. The odds of placental abnormality were significantly increased when CPR < 5^th^ centile (aOR, 3.17 (95% CI, 1.95–5.16); *P* < 0.001), abnormal UA Doppler (aOR, 2.97 (95% CI, 1.80–4.90); *P* < 0.001) and mean UtA‐PI > 95^th^ centile (aOR, 5.42 (95% CI, 2.75–10.70); *P* < 0.001) were present (Table 3, Figure S1). The odds of MVM were also significantly higher when CPR < 5^th^ centile (aOR, 2.47 (95% CI, 1.64–4.33); *P* < 0.001), abnormal UA Doppler (aOR, 3.13 (95% CI, 1.91–5.12); *P* < 0.001) and mean UtA‐PI > 95^th^ centile (aOR, 4.01 (95% CI, 2.25–7.13); *P* < 0.001) were present. The odds of placental abnormality were almost four times higher in women with PlGF < 100 ng/L (aOR, 3.66 (95% CI, 2.22–6.06); *P* < 0.001) or an elevated sFlt‐1/PlGF ratio (aOR, 3.74 (95% CI, 2.17–6.43); *P* < 0.001), compared to controls. Similarly, the odds of MVM were almost three times higher in women with PlGF < 100 ng/L (aOR, 2.89 (95% CI, 1.72–4.85); *P* = 0.001) or an elevated sFlt‐1/PlGF ratio (aOR, 3.15 (95% CI, 1.83–5.45); *P* < 0.001).

**Table 3:** Multivariable logistic regression analysis of effect of fetoplacental Doppler parameters on placental abnormality in pregnancies with small‐for‐gestational‐age fetus

Outcome/model	Univariable OR	*P*	Multivariable aOR*	*P*
*Outcome: any placental abnormality*				
Model 1: *Z*‐scores				
CPR	0.44 (0.33–0.58)	< 0.001	0.52 (0.39–0.69)	< 0.001
UA‐PI	3.98 (2.58–6.15)	< 0.001	3.26 (2.07–5.14)	< 0.001
Mean UtA‐PI	2.65 (2.05–3.43)	< 0.001	2.21 (1.66–2.95)	< 0.001
PlGF level	0.54 (0.32–0.93)	0.027	0.69 (0.41–1.14)	0.145
sFlt‐1/PlGF ratio	28.06 (3.44–229.11)	0.002	17.79 (2.06–153.81)	0.009
Model 2: Cut‐off points				
CPR ≥ 5^th^ centile	Reference		Reference	
CPR < 5^th^ centile	4.38 (2.74–6.99)	< 0.001	3.17 (1.95–5.16)	< 0.001
UA‐PI ≤ 95^th^ centile	Reference		Reference	
UA‐PI > 95^th^ centile or AREDF	4.13 (2.57–6.64)	< 0.001	2.97 (1.80–4.90)	< 0.001
Mean UtA‐PI ≤ 95^th^ centile	Reference		Reference	
Mean‐UtA PI > 95^th^ centile	7.86 (4.09–15.07)	< 0.001	5.42 (2.75–10.70)	< 0.001
PlGF ≥ 100 ng/L	Reference		Reference	
PlGF < 100 ng/L	5.15 (3.26–8.13)	< 0.001	3.66 (2.22–6.06)	< 0.001
sFlt‐1/PlGF ratio ≤ 5.78 if GA < 28 weeks or ≤ 38 if GA ≥ 28 weeks	Reference		Reference	
sFlt‐1/PlGF ratio > 5.78 if GA < 28 weeks or > 38 if GA ≥ 28 weeks	4.91 (2.99–8.06)	< 0.001	3.74 (2.17–6.43)	< 0.001
*Outcome: maternal vascular malperfusion*				
Model 3: *Z*‐scores				
CPR	0.41 (0.31–0.56)	< 0.001	0.54 (0.39–0.73)	< 0.001
UA‐PI	4.24 (2.82–6.35)	< 0.001	3.19 (2.08–4.89)	< 0.001
Mean UtA‐PI	2.97 (2.24–3.95)	< 0.001	2.32 (1.70–3.17)	< 0.001
PlGF level	0.28 (0.14–0.58)	0.001	0.44 (0.23–0.83)	0.011
sFlt‐1/PlGF ratio	9.24 (2.49–34.28)	0.001	4.50 (1.39–14.58)	0.012
Model 4: Cut‐off points				
CPR ≥ 5^th^ centile	Reference		Reference	
CPR < 5^th^ centile	4.12 (2.65–6.43)	< 0.001	2.47 (1.64–4.33)	< 0.001
UA‐PI ≤ 95^th^ centile	Reference		Reference	
UA‐PI > 95^th^ centile or AREDF	4.70 (2.99–7.40)	< 0.001	3.13 (1.91–5.12)	< 0.001
Mean UtA‐PI ≤ 95^th^ centile	Reference		Reference	
Mean‐UtA PI > 95^th^ centile	6.49 (3.80–11.08)	< 0.001	4.01 (2.25–7.13)	< 0.001
PlGF ≥ 100 ng/L	Reference		Reference	
PlGF < 100 ng/L	4.87 (3.08–7.72)	< 0.001	2.89 (1.72–4.85)	< 0.001
sFlt‐1/PlGF ratio ≤ 5.78 if GA < 28 weeks or ≤ 38 if GA ≥ 28 weeks	Reference		Reference	
sFlt‐1/PlGF ratio > 5.78 if GA < 28 weeks or > 38 if GA ≥ 28 weeks	4.94 (3.00–8.13)	< 0.001	3.15 (1.83–5.45)	< 0.001

Values in parentheses are 95% CI.

*Z*‐scores were analyzed per unit increase above the mean.

Odds ratios (ORs) and adjusted ORs (aORs) with lower values (< 1) indicate lower odds of having the pathology (as these are below the mean).

*P* < 0.05 considered statistically significant.

* Adjusted for pre‐eclampsia.

AREDF, absent or reversed end‐diastolic flow; CPR, cerebroplacental ratio; GA, gestational age; PI, pulsatility index; PlGF, placental growth factor; sFlt‐1, soluble fms‐like tyrosine kinase‐1; UA, umbilical artery; UtA, uterine artery.

Table S2 lists pregnancy outcomes stratified by fetoplacental Doppler parameters and placental biomarkers. The median gestational age at delivery and birth weight were significantly lower in all cases with abnormal fetoplacental Doppler parameters or placental biomarkers compared to their control groups with normal corresponding parameters, and particularly low when mean UtA‐PI > 95^th^ centile (34 (IQR, 31–37) weeks *vs* 36 (IQR, 34–38) weeks (*P* < 0.001) and 1543 (IQR, 1020–2100) g *vs* 1978 (IQR, 1570–2510) g (*P* < 0.001), respectively). All cases of stillbirth and neonatal mortality had abnormal placental biomarkers. Women with CPR < 5^th^ centile, UA Doppler abnormality, mean UtA‐PI > 95^th^ centile, PlGF < 100 ng/mL or an elevated sFlt‐1/PlGF ratio had a significantly increased risk of PTB, especially medically indicated PTB, as well as Cesarean section (both emergency and elective), emergency operative delivery for non‐reassuring fetal status and an infant with severe non‐neurological morbidity, compared to controls with normal corresponding parameters.

## DISCUSSION

### Principal findings

In this prospective study we have shown that mean UtA‐PI > 95^th^ centile, CPR < 5^th^ centile, abnormal UA Doppler (UA‐PI > 95^th^ centile or AREDF), PlGF level < 100 ng/L and an elevated sFlt‐1/PlGF ratio (> 5.78 if gestational age < 28 weeks or > 38 if gestational age ≥ 28 weeks) are all strongly associated with placental abnormality, particularly MVM lesions. Our results suggest that mean UtA‐PI > 95^th^ centile has the strongest association with placental abnormality, particularly MVM. Our findings imply that all of these fetoplacental Doppler and placental biomarker abnormalities are independently associated with the presence of typical placental abnormalities, that are in turn associated with placental dysfunction. Abnormal fetoplacental Doppler indices and placental biomarkers were also strongly associated with lower median placental weight, lower median gestational age at birth, lower birth weight, overall PTB and medically indicated PTB, Cesarean section, emergency operative delivery for non‐reassuring fetal status and severe neonatal non‐neurological morbidity.

### Clinical implications

Our results are consistent with a retrospective study of Ashwal *et al*.^19^ which found that, among SGA fetuses, the combination of UA and MCA Doppler indices was accurate in ruling out FGR due to MVM, but of limited value in excluding non‐MVM pathology. Similarly, Paules *et al*.^31^ demonstrated that abnormal UtA, UA and MCA‐PI and CPR were significantly associated with MVM, but not with FVM or other types of placental abnormality, in SGA and pre‐eclamptic pregnancies. In another smaller cohort study of late FGR pregnancies^52^, CPR < 5^th^ centile was found to be reflective of both MVM and FVM features, with lower mean placental weight in late‐onset FGR. Spinillo *et al*.^32^ found that lower CPR centiles were associated with placental MVM features in FGR pregnancies (OR, 2.0 (95% CI, 1.07–3.71); *P* = 0.03), and histological evidence of placental occlusion was more commonly seen in early‐onset disease. More recently, Shmueli *et al*.^52^ reported that CPR < 5^th^ centile in late FGR pregnancies was associated with higher risk for placental MVM (OR, 2.17 (95% CI, 1.63–4.19)) and FVM lesions (OR, 1.31 (95% CI, 1.09–3.97)) when compared to FGR placentae with normal CPR.

A recent study of Agrawal *et al*.^21^ reported that placentae with MVM features were associated with decreased maternal PlGF levels and abnormal mean UtA‐PI, finding that the mean UtA‐PI was highly abnormal in the MVM cohort but not in the non‐MVM group. They also demonstrated that adding 4‐weekly serial PlGF measurements between 16 and 36 weeks of gestation performed better than using UtA Doppler assessment alone to identify women at risk of adverse perinatal outcome: 28/29 (96.5%) women who experienced stillbirth had one or more low PlGF level result compared to 21/29 (72.4%) who experienced stillbirth and had abnormal UtA Doppler. This is broadly consistent with our data demonstrating that low maternal PlGF levels (< 100 ng/L), an elevated sFlt‐1/PlGF ratio and abnormal fetoplacental Doppler were strongly associated with SGA placentae with MVM features. However, we also showed that some of these measurements were strongly associated with other placental abnormalities (FVM, VUE and CHI) and adverse pregnancy outcomes. Our data suggest that, of all the fetoplacental Doppler indices and placental biomarkers investigated, mean UtA‐PI > 95^th^ centile has the strongest association with placental abnormality, particularly MVM. Our findings thus support the rationale of ISUOG^38^ and other recommendations^53^, ^54^ for UtA Doppler assessment in cases of suspected FGR to ascertain the etiology underlying the abnormal fetal size and growth.

Current recommendations^55^, ^56^, ^57^ suggest commencing administration of low‐dose aspirin prophylaxis before 16 weeks' gestation for women at risk of pre‐eclampsia or placental dysfunction, and a recent systematic review and meta‐analysis^58^ found that low molecular weight heparin (LMWH) therapy started before 16 weeks is associated with a significant reduction in the risk of these complications. LMWH promotes release of PlGF^59^ from endothelial cells and increases PlGF levels in women with pre‐eclampsia.^60^ McLaughin *et al*.^60^ demonstrated that the addition of LMWH (in women already taking aspirin prophylaxis) in the early second trimester results in restoration of PlGF levels, which mediates improved perinatal outcomes: later mean gestation at birth (36 (IQR, 33–37) weeks *vs* 28 (IQR, 27–31) weeks) and higher birth weight (1.93 (IQR, 1.1–2.7) kg *vs* 0.73 (IQR, 0.52–1.03) kg). In our study, 22% of women were taking aspirin prophylaxis and 6% had LMWH therapy, and the rates in subgroups according to fetoplacental Doppler parameter or placental biomarker abnormality were highest amongst the subgroup of women with mean UtA‐PI > 95^th^ centile.

### Strengths and limitations

Strengths of our study include its prospective nature and the large number of well‐characterized SGA pregnancies with detailed placental histopathology. There is a paucity of data investigating the associations between fetoplacental Doppler parameters and placental biomarkers with specific placental abnormalities. Previous reports have either been limited to case studies^60^, ^61^ or focused on PlGF levels alone^21^, without incorporating its antiangiogenic counterpart (sFlt‐1 and the sFlt‐1/PlGF ratio), or focused mainly on the association of fetoplacental Doppler abnormalities with MVM and FVM lesions^52^, ^62^. In our study, perinatal pathologists undertaking the placental examinations were blinded to the ultrasound findings and placental biomarker results, and adhered strictly to the Amsterdam criteria for reporting placental lesions^41^. All ultrasound examinations were performed by experienced obstetric sonographers and reported by maternal–fetal medicine specialists who were also blinded to the placental biomarker levels. Our analyses were also adjusted for pre‐eclampsia to reduce the potential confounding effect on the study outcomes. The limitations of our study include selection bias, potentially compromising the generalizability of this study. We were unable to perform multivariable logistic analyses for non‐MVM placental abnormalities because of the low number of cases in this study cohort.

### Conclusions

Our findings provide further evidence of the association and clinical utility of fetoplacental Doppler parameters and placental biomarkers with specific placental pathology known to result in placental dysfunction in SGA pregnancies. Mean UtA‐PI > 95^th^ centile had the highest association with placental abnormality, particularly MVM. Furthermore, our results also show that a low CPR (< 5^th^ centile) is as good as the more conventional fetoplacental Doppler parameters and placental biomarkers in predicting placental abnormality, further supporting its role as a reliable prenatal indicator of FGR secondary to placental dysfunction.

## Supporting information


**Table S1** Comparison of placental pathological features in SGA pregnancies, stratified by fetoplacental Doppler parameters and placental biomarkers

**Table S2** Pregnancy outcomes stratified by fetoplacental Doppler parameters and placental biomarkers

**Figure S1** Box‐and‐whiskers plots of raw values of fetoplacental Doppler parameters (cerebroplacental ratio (CPR), umbilical artery pulsatility index (PI) and mean uterine artery PI) and placental biomarkers (placental growth factor (PlGF) and soluble fms‐like tyrosine kinase‐1 (sFlt‐1)/PlGF ratio) at last measurement before birth in pregnancies with small‐for‐gestational‐age fetus, stratified by the presence or absence of any placental abnormality and of maternal vascular malperfusion (MVM). Boxes show median and interquartile range (IQR), whiskers show 1.5 × interquartile range (IQR) and circles are outliers.

## Data Availability

The data that support the findings of this study are available on request from the corresponding author. The data are not publicly available due to privacy or ethical restrictions.
