# Supplementary material for: Placental biomarker and fetoplacental Doppler abnormalities are strongly associated with placental pathology in pregnancies with small‐for‐gestational‐age fetus: prospective study
Source: Ultrasound Obstet Gynecol. 2025 May 7;65(6):749–60. doi: 10.1002/uog.29237 (PMC12127712; doi:10.1002/uog.29237)
Supplement: Supplementary file 1 — Table S1 Comparison of placental pathological features in SGA pregnancies, stratified by fetoplacental Doppler parameters and placental biomarkers [file UOG-65-749-s002.docx]

**Table S1** Comparison of placental pathological features in SGA pregnancies, stratified by fetoplacental Dopplers parameters and placental biomarkers

| **Placental Features** | **Total** | **CPR <5^th^ centile** | **P value** | **Abnormal UA Dopplers^a^** | **P value** | **Mean UtA PI >95^th^ centile** | **P value** | **PlGF <100 ng/L** | **P value** | **High sFlt-1/PlGF ratio^b^** | **P value** |
| --- | --- | --- | --- | --- | --- | --- | --- | --- | --- | --- | --- |
|  | **N=367** | **N=84** |  | **N=139** |  | **N=94** |  | **N=181** |  | **N=141** |  |
| Placental weight (grams) | 332 (268, 386) | 272 (198, 330) | <0.001 | 262 (184, 317) | <0.001 | 258 (177, 330) | <0.001 | 280 (201, 355) | <0.001 | 284 (204, 359) | <0.001 |
| Placental weight centile |  |  |  |  |  |  |  |  |  |  |  |
| <3 | 303 (82.6%) | 135 (91.2%) | <0.001 | 124 (89.2%) | <0.001 | 89 (94.7%) | <0.001 | 156 (86.2%) | <0.001 | 122 (86.5%) | <0.001 |
| 3 to <10 | 48 (13.1%) | 10 (6.8%) | 0.003 | 14 (10.1%) | 0.125 | 2 (2.1%) | 0.002 | 17 (9.4%) | 0.029 | 12 (8.5%) | 0.O51 |
| 10 to <25 | 11 (3.0%) | 3 (2.0%) | 0.268 | 1 (0.7%) | 0.067 | 1 (1.1%) | 0.178 | 5 (2.8%) | 0.597 | 4 (2.8%) | 0.588 |
| 25 to <50 | 4 (1.1%) | 0 (0.0%) |  | 0 (0.0%) |  | 2 (2.1%) | 0.385 | 3 (1.7%) | 0.412 | 3 (2.1%) | 0.324 |
| >75 | 1 (0.3%) | 0 (0.0%) |  | 0 (0.0%) |  | 0 (0.0%) |  | 0 (0.0%) |  | 0 (0.0%) |  |
| Placental thickness (mm) | 20 (15, 25) | 18 (15, 24) | <0.001 | 17 (14, 20) | <0.001 | 20 (15, 23) | 0.089 | 20 (15, 24) | 0.013 | 20 (15, 24) | 0.077 |
| Cord insertion site |  |  |  |  |  |  |  |  |  |  |  |
| Central/Paracentral | 299 (81.5%) | 116 (78.4%) |  | 110 (79.1%) |  | 74 (78.7%) |  | 145 (80.1%) |  | 113 (80.1%) |  |
| Marginal | 63 (17.2%) | 30 (20.3%) | 0.197 | 27 (19.4%) | 0.368 | 19 (20.2%) | 0.373 | 32 (17.7%) | 0.989 | 24 (17.0%) | 0.892 |
| Velamentous | 5 (1.4%) | 2 (1.4%) | 0.956 | 2 (1.4%) | 0.883 | 1 (1.1%) | 0.808 | 4 (2.2%) | 0.227 | 4 (2.8%) | 0.174 |
| Cord coiling |  |  |  |  |  |  |  |  |  |  |  |
| Normal | 281 (76.8%) | 120 (81.1%) |  | 112 (80.6%) |  | 77 (81.9%) |  | 139 (76.8%) |  | 111 (78.7%) |  |
| Hypocoiled | 32 (8.7%) | 11 (7.4%) | 0.368 | 11 (7.9%) | 0.549 | 7 (7.4%) | 0.506 | 14 (7.7%) | 0.399 | 9 (6.4%) | 0.406 |
| Hypercoiled | 53 (14.5%) | 17 (11.5%) | 0.152 | 16 (11.5%) | 0.187 | 10 (10.6%) | 0.198 | 28 (15.5%) | 0.774 | 21 (14.9%) | 0.570 |
| True Knots | 10 (2.7%) | 3 (2.0%) | 0.500 | 3 (2.2%) | 0.601 | 2 (2.1%) | 0.678 | 6 (3.3%) | 0.578 | 6 (4.3%) | 0.240 |
| Single Umbilical Artery | 10 (2.7%) | 3 (2.0%) | 0.500 | 4 (2.9%) | 0.894 | 2 (2.1%) | 0.678 | 4 (2.2%) | 0.679 | 3 (2.1%) | 0.594 |
| **MVM** | 159 (43.3%) | 94 (63.5%) | <0.001 | 92 (66.2%) | <0.001 | 71 (75.5%) | <0.001 | 109 (60.2%) | <0.001 | 84 (59.6%) | <0.001 |
| Grading |  |  |  |  |  |  |  |  |  |  |  |
| Mild (focal) | 74 (46.5%) | 29 (30.9%) |  | 31 (33.7%) |  | 22 (31.0%) |  | 37 (33.9%) |  | 34 (40.5%) |  |
| Severe (diffuse) | 85 (53.5%) | 65 (69.1%) | <0.001 | 61 (66.3%) | <0.001 | 49 (69.0%) | <0.001 | 72 (66.1%) | <0.001 | 50 (59.5%) | 0.000 |
| Placental hypoplasia | 192 (52.3%) | 97 (65.5%) | <0.001 | 94 (67.6%) | <0.001 | 66 (70.2%) | <0.001 | 112 (61.9%) | <0.001 | 87 (61.7%) | 0.012 |
| Placental infarction | 96 (26.2%) | 62 (41.9%) | <0.001 | 56 (40.3%) | <0.001 | 49 (52.1%) | <0.001 | 69 (38.1%) | <0.001 | 59 (41.8%) | <0.001 |
| Retroplacental hemorrhage | 28 (7.6%) | 16 (10.8%) | 0.064 | 16 (11.5%) | 0.033 | 11 (11.7%) | 0.090 | 21 (11.6%) | 0.004 | 15 (10.6%) | 0.042 |
| Distal villous hypoplasia | 121 (33.0%) | 76 (51.4%) | <0.001 | 72 (51.8%) | <0.001 | 52 (55.3%) | <0.001 | 87 (48.1%) | <0.001 | 68 (48.2%) | <0.001 |
| Accelerated villous maturation | 128 (34.9%) | 82 (55.4%) | <0.001 | 79 (56.8%) | <0.001 | 59 (62.8%) | <0.001 | 92 (50.8%) | <0.001 | 70 (49.6%) | <0.001 |
| Syncytial knots | 83 (22.6%) | 55 (37.2%) | <0.001 | 49 (35.3%) | <0.001 | 40 (42.6%) | <0.001 | 61 (33.7%) | <0.001 | 48 (34.0%) | <0.001 |
| Perivillous fibrin deposition | 57 (15.5%) | 38 (25.7%) | <0.001 | 37 (26.6%) | <0.001 | 31 (33.0%) | <0.001 | 41 (22.7%) | 0.000 | 33 (23.4%) | <0.001 |
| Villous agglutination | 24 (6.5%) | 17 (11.5%) | 0.052 | 16 (11.5%) | 0.039 | 19 (20.2%) | <0.001 | 17 (9.4%) | <0.001 | 12 (8.5%) | <0.001 |
| Decidual arteriopathy | 36 (9.8%) | 26 (17.6%) | <0.001 | 23 (16.5%) | 0.001 | 21 (22.3%) | <0.001 | 29 (16.0%) | 0.000 | 20 (14.2%) | 0.000 |
| Insufficient vessel remodeling | 32 (8.7%) | 26 (17.6%) | <0.001 | 24 (17.3%) | <0.001 | 17 (18.1%) | 0.000 | 27 (14.9%) | 0.000 | 18 (12.8%) | 0.002 |
| Fibrinoid necrosis | 22 (6.0%) | 13 (8.8%) | 0.071 | 12 (8.6%) | 0.103 | 16 (17.0%) | <0.001 | 16 (8.8%) | 0.013 | 9 (6.4%) | 0.109 |
| **FVM** | 20 (5.4%) | 12 (8.1%) | 0.072 | 10 (7.2%) | 0.256 | 9 (9.6%) | 0.048 | 14 (7.7%) | 0.052 | 10 (7.1%) | 0.307 |
| Grading |  |  |  |  |  |  |  |  |  |  |  |
| Low (segmental) | 16 (80.0%) | 11 (91.7%) |  | 8 (80.0%) |  | 7 (77.8%) |  | 11 (78.6%) |  | 8 (80.0%) |  |
| High (global) | 4 (20.0%) | 1 (8.3%) | 0.149 | 2 (20.0%) | 1.000 | 2 (22.2%) | 0.827 | 3 (21.4%) | 0.948 | 2 (20.0%) | 0.692 |
| Thrombosis | 14 (3.8%) | 8 (5.4%) | 0.200 | 7 (5.0%) | 0.346 | 8 (8.5%) | 0.011 | 10 (5.5%) | 0.133 | 6 (4.3%) | 0.815 |
| Avascular villi | 18 (4.9%) | 9 (6.1%) | 0.395 | 9 (6.5%) | 0.282 | 9 (9.6%) | 0.020 | 11 (6.1%) | 0.258 | 9 (6.4%) | 0.295 |
| Intramural fibrin deposition | 8 (2.2%) | 3 (2.0%) | 0.869 | 4 (2.9%) | 0.480 | 3 (3.2%) | 0.443 | 5 (2.8%) | 0.519 | 5 (3.5%) | 0.205 |
| Villous stromal-vascular karyorrhexis | 9 (2.5%) | 5 (3.4%) | 0.354 | 4 (2.9%) | 0.682 | 3 (3.2%) | 0.594 | 6 (3.3%) | 0.192 | 7 (5.0%) | 0.048 |
| Stem vessel obliteration | 9 (2.5%) | 5 (3.4%) | 0.354 | 5 (3.6%) | 0.279 | 3 (3.2%) | 0.594 | 7 (3.9%) | 0.127 | 6 (4.3%) | 0.236 |
| **VUE** | 49 (13.4%) | 28 (18.9%) | 0.011 | 25 (18.0%) | 0.044 | 18 (19.1%) | 0.058 | 33 (18.2%) | 0.010 | 30 (21.3%) | 0.003 |
| Grading |  |  |  |  |  |  |  |  |  |  |  |
| Low | 22 (44.9%) | 11 (39.3%) |  | 11 (44.0%) |  | 8 (44.4%) |  | 12 (36.4%) |  | 12 (40.0%) |  |
| High | 27 (55.1%) | 17 (60.7%) | 0.368 | 14 (56.0%) | 0.898 | 10 (55.6%) | 0.962 | 21 (63.6%) | 0.135 | 18 (60.0%) | 0.296 |
| Lymphohistiocytic infiltration | 49 (13.4%) | 27 (18.2%) | 0.026 | 24 (17.3%) | 0.088 | 17 (18.1%) | 0.121 | 33 (18.2%) | 0.010 | 30 (21.3%) | 0.003 |
| Chronic plasma cell deciduitis | 8 (2.2%) | 5 (3.4%) | 0.212 | 4 (2.9%) | 0.480 | 3 (3.2%) | 0.443 | 5 (2.8%) | 0.520 | 4 (2.8%) | 0.336 |
| **DVM** | 19 (5.2%) | 3 (2.0%) | 0.036 | 4 (2.9%) | 0.132 | 4 (4.3%) | 0.641 | 10 (5.5%) | 0.893 | 4 (2.8%) | 0.060 |
| Grading |  |  |  |  |  |  |  |  |  |  |  |
| Focal | 10 (52.6%) | 2 (66.7%) |  | 3 (75.0%) |  | 3 (75.0%) |  | 7 (70.0%) |  | 4 (100.0%) |  |
| Diffuse | 9 (47.4%) | 1 (33.3%) | 0.610 | 1 (25.0%) | 0.343 | 1 (25.0%) | 0.343 | 3 (30.0%) | 0.129 | 0 (0.0%) | - |
| **CHI** | 6 (1.6%) | 3 (2.0%) | 0.629 | 3 (2.2%) | 0.542 | 2 (2.1%) | 0.665 | 6 (3.3%) | <0.001 | 5 (3.5%) | 0.108 |
| **Chorangiosis** | 2 (0.5%) | 0 (0.0%) | - | 0 (0.0%) | - | 0 (0.0%) | - | 2 (1.1%) | 0.103 | 2 (1.4%) | 0.108 |
| **Chorangioma** | 4 (1.1%) | 1 (0.7%) | 0.032 | 1 (0.7%) | 0.031 | 1 (1.1%) | 0.304 | 3 (1.7%) | 0.100 | 3 (2.1%) | 0.340 |
| **Other pathology^c^** | 73 (19.9%) | 20 (13.5%) | 0.013 | 20 (14.4%) | 0.041 | 14 (14.9%) | 0.162 | 36 (19.9%) | 0.830 | 31 (22.0%) | 0.753 |
| **Breus mole** | 2 (0.5%) | 0 (0.0%) | - | 0 (0.0%) | - | 0 (0.0%) | - | 2 (1.1%) | 0.100 | 1 (0.7%) | 0.925 |

Data are presented median (interquartile range) for continuous measures, and n (%) for categorical measures. P value generated by logistic regression.

SGA – Small for gestational age; MVM – Maternal vascular malperfusion; FVM – Fetal vascular malperfusion; VUE – Villitis of unknown etiology; DVM – Delayed villous maturation; CHI - chronic histiocytic intervillositis.

^a^ Abnormal UA included UA PI >95^th^ centile and absent or reversed end diastolic flow.

^b^ sFlt-1/PlGF ratio >5.78 if <28 weeks or >38 if ≥28 weeks of gestation.

^c^ Ascending intrauterine infection, subchorionic or intraparenchymal hemorrhage, and villous hydrops of uncertain significance.
