# Supplementary material for: Placental biomarker and fetoplacental Doppler abnormalities are strongly associated with placental pathology in pregnancies with small‐for‐gestational‐age fetus: prospective study
Source: Ultrasound Obstet Gynecol. 2025 May 7;65(6):749–60. doi: 10.1002/uog.29237 (PMC12127712; doi:10.1002/uog.29237)
Supplement: Supplementary file 2 — Table S2 Pregnancy outcomes stratified by fetoplacental Doppler parameters and placental biomarkers [file UOG-65-749-s003.docx]

**Table S2** Pregnancy outcomes stratified by fetoplacental Dopplers and placental biomarkers

| **Outcome** | **Total** | **CPR <5^th^ centile** | **P value** | **Abnormal UA Dopplers^a^** | **P value** | **Mean UtA PI >95^th^ centile** | **P value** | **PlGF level**  **<100 ng/L** | **P value** | **High**  **sFlt-1/PlGF ratio^b^** | **P value** |
| --- | --- | --- | --- | --- | --- | --- | --- | --- | --- | --- | --- |
|  | **N=367** | **N=148** |  | **N=139** |  | **N=94** |  | **N=181** |  | **N=141** |  |
| GA at delivery (weeks) | 37 (35, 38) | 36 (32, 37) | <0.001 | 35 (31, 37) | <0.001 | 34 (31, 37) | <0.001 | 36 (32, 37) | <0.001 | 36 (33, 37) | <0.001 |
| Birthweight (grams) | 2260 (1740, 2550) | 1780 (1208, 2190) | <0.001 | 1700 (1059, 2190) | <0.001 | 1543 (1020, 2100) | <0.001 | 1800 (1193, 2210) | <0.001 | 1870 (1350, 2294) | <0.001 |
| Birthweight centiles |  |  |  |  |  |  |  |  |  |  |  |
| <1^st^ | 68 (18.5%) | 33 (22.3%) | 0.129 | 30 (21.6%) | 0.241 | 24 (25.5%) | 0.045 | 42 (23.2%) | 0.017 | 37 (26.2%) | 0.007 |
| <3^rd^ | 152 (41.4%) | 69 (46.6%) | 0.097 | 65 (46.8%) | 0.101 | 45 (47.9%) | 0.142 | 83 (45.9%) | 0.115 | 70 (49.6%) | 0.133 |
| <10^th^ | 300 (81.7%) | 123 (83.1%) | 0.579 | 115 (82.7%) | 0.702 | 78 (83.0%) | 0.720 | 153 (84.5%) | 0.153 | 121 (85.8%) | 0.194 |
| Infant sex |  |  |  |  |  |  |  |  |  |  |  |
| Male | 164 (44.7%) | 71 (48.0%) |  | 71 (51.1%) |  | 45 (47.9%) |  | 85 (47.0%) |  | 66 (46.8%) |  |
| Female | 202 (55.0%) | 77 (52.0%) | 0.317 | 68 (48.9%) | 0.060 | 49 (52.1%) | 0.489 | 95 (52.5%) | 0.535 | 74 (52.5%) | 0.525 |
| Stillbirth | 4 (1.1%) | 2 (1.4%) | 0.694 | 3 (2.2%) | 0.165 | 3 (3.2%) | 0.059 | 4 (2.2%) | - | 4 (2.8%) | - |
| All preterm birth (PTB) | 127 (34.6%) | 91 (61.5%) | <0.001 | 86 (61.9%) | <0.001 | 63 (67.0%) | <0.001 | 108 (59.7%) | <0.001 | 78 (55.3%) | <0.001 |
| Spontaneous PTB <34 weeks | 1 (0.3%) | 0 (0.0%) | - | 0 (0.0%) | - | 0 (0.0%) | - | 0 (0.0%) | - | 0 (0.0%) | - |
| All Spontaneous PTB | 13 (3.5%) | 3 (2.0%) | 0.210 | 2 (1.4%) | 0.109 | 1 (1.1%) | 0.166 | 8 (4.4%) | 0.448 | 8 (5.7%) | 0.034 |
| Medically indicated PTB | 116 (31.6%) | 89 (60.1%) | <0.001 | 84 (60.4%) | <0.001 | 64 (68.1%) | <0.001 | 101 (55.8%) | <0.001 | 71 (50.4%) | <0.001 |
| Mode of delivery |  |  |  |  |  |  |  |  |  |  |  |
| Spontaneous vaginal | 128 (34.9%) | 26 (17.6%) |  | 19 (13.7%) |  | 16 (17.0%) |  | 34 (18.8%) |  | 31 (22.0%) |  |
| Instrumental vaginal | 15 (4.1%) | 4 (2.7%) | 0.570 | 4 (2.9%) | 0.247 | 0 (0.0%) | - | 5 (2.8%) | 0.632 | 3 (2.1%) | 0.531 |
| Elective Cesarean | 93 (25.3%) | 42 (28.4%) | 0.0001 | 47 (33.8%) | <0.001 | 30 (31.9%) | 0.0005 | 49 (27.1%) | 0.0001 | 38 (27.0%) | 0.002 |
| Emergency Cesarean | 131 (35.7%) | 76 (51.4%) | <0.001 | 69 (49.6%) | <0.001 | 48 (51.1%) | <0.001 | 93 (51.4%) | <0.001 | 69 (48.9%) | <0.001 |
| Emergency operative delivery for NRFS | 86 (23.4%) | 56 (37.8%) | <0.001 | 51 (36.7%) | <0.001 | 34 (36.2%) | 0.0009 | 62 (34.3%) | <0.001 | 46 (32.6%) | <0.001 |
| Neonatal mortality | 3 (0.8%) | 2 (1.4%) | 0.374 | 3 (2.2%) | - | 2 (2.1%) | 0.149 | 3 (1.7%) | - | 3 (2.13%) | - |
| Severe neurological morbidity ^c^ | 12 (3.3%) | 6 (4.1%) | 0.491 | 7 (5.0%) | 0.149 | 5 (5.3%) | 0.206 | 11 (6.1%) | 0.022 | 6 (4.3%) | 0.071 |
| Severe non-neurological morbidity ^d^ | 124 (33.8%) | 78 (52.7%) | <0.001 | 77 (55.4%) | <0.001 | 52 (55.3%) | <0.001 | 91 (50.3%) | <0.001 | 66 (46.8%) | <0.001 |

Data are presented as median (interquartile range) for continuous measures and n (%) for categorical measures. P value generated by logistic regression. Significant p value <0.05.

CPR – Cerebroplacental ratio; UA – Umbilical artery; UtA PI – Uterine artery pulsatility index; ; PlGF – Placental growth factor; sFlt-1 – Soluble fms-like tyrosine kinase-1; GA – Gestational age; PTB – Preterm birth; NRFS – Non-reassuring fetal status.

^a^ Abnormal UA included UA PI >95th centile and absent or reversed end diastolic flow. ^b^ sFlt-1/PlGF ratio >5.78 if <28 weeks or >38 if ≥28 weeks of gestation.

^c^ Hypoxic-ischemic encephalopathy, Intraventricular hemorrhage, and seizure.

^d^ NICU admission ≥ 24 hours, neonatal sepsis, necrotizing enterocolitis, respiratory distress syndrome, and anemia requiring blood transfusion.
