# Supplementary material for: Placental biomarker and fetoplacental Doppler abnormalities are strongly associated with placental pathology in pregnancies with small‐for‐gestational‐age fetus: prospective study
Source: Ultrasound Obstet Gynecol. 2025 May 7;65(6):749–60. doi: 10.1002/uog.29237 (PMC12127712; doi:10.1002/uog.29237)
Supplement: Supplementary file 3 — Figure S1 Box‐and‐whiskers plots of raw values of fetoplacental Doppler parameters (cerebroplacental ratio (CPR), umbilical artery pulsatility index (PI) and mean uterine artery PI) and placental biomarkers (placental growth factor (PlGF) and soluble fms‐like tyrosine kinase‐1 (sFlt‐1)/PlGF ratio) at last measurement before birth in pregnancies with small‐for‐gestational‐age fetus, stratified by the presence or absence of any placental abnormality and of maternal vascular malperfusion (MVM). Boxes show median and interquartile range (IQR), whiskers show 1.5 × interquartile range (IQR) and circles are outliers. [file UOG-65-749-s001.docx]

| 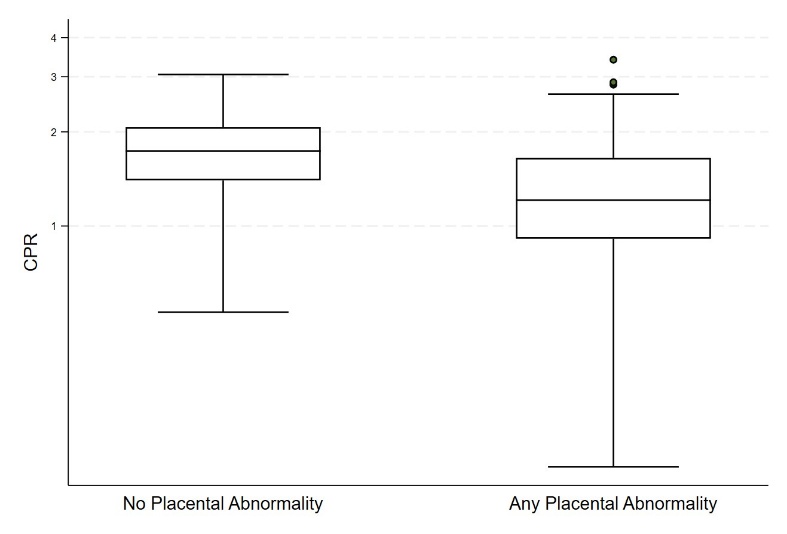 | 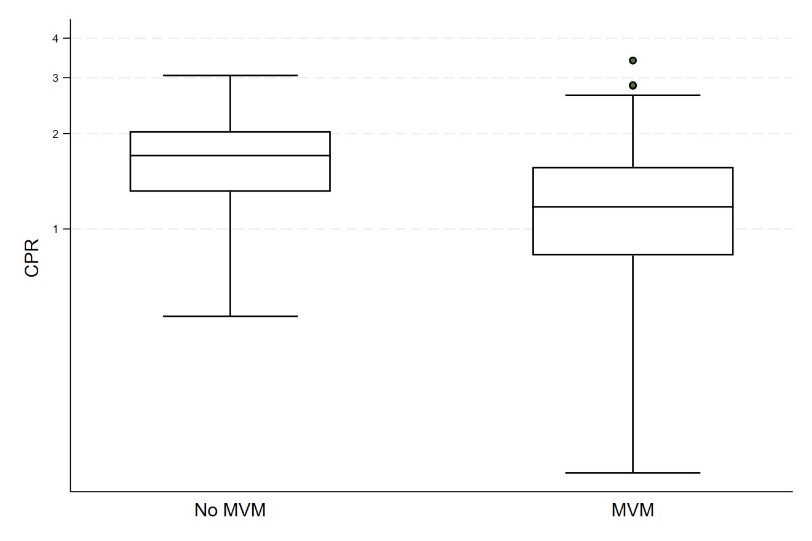 |
| --- | --- |
| 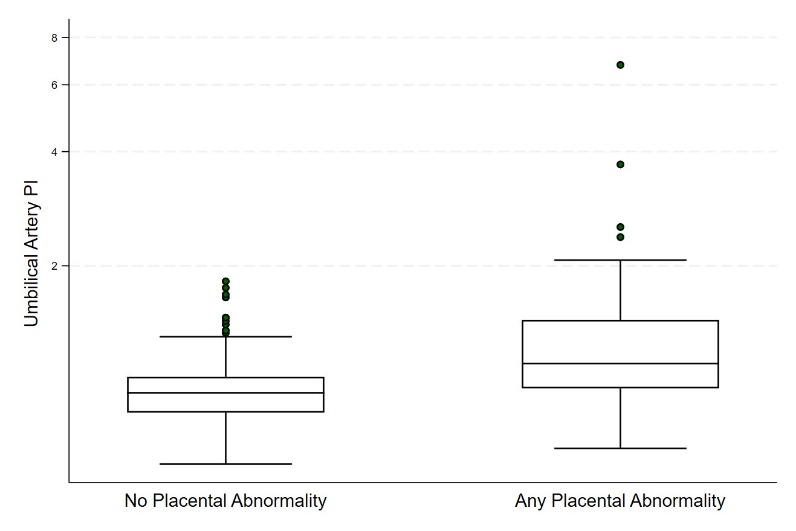 | 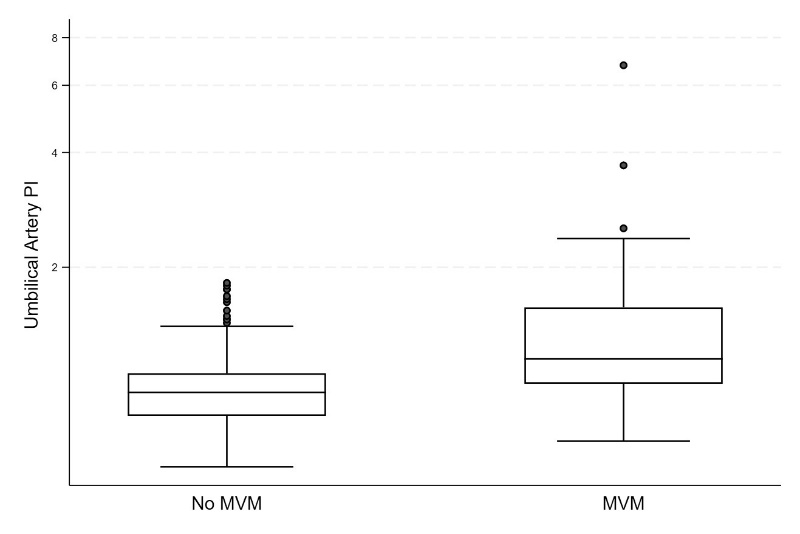 |
| 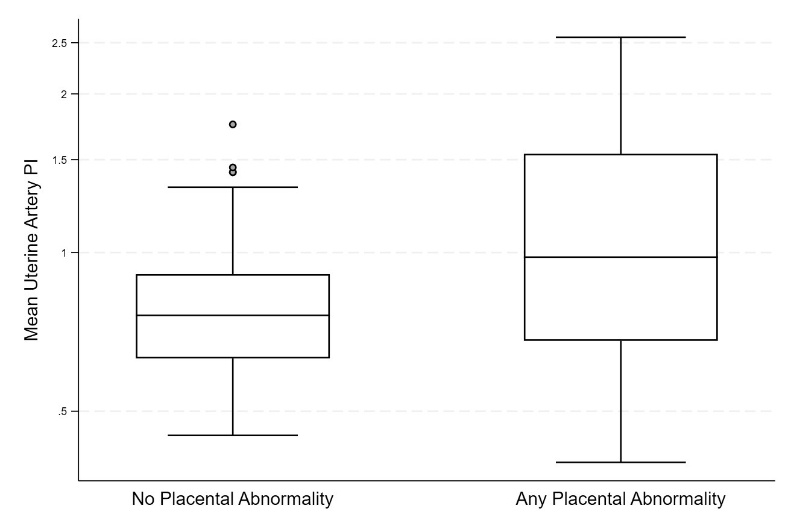 | 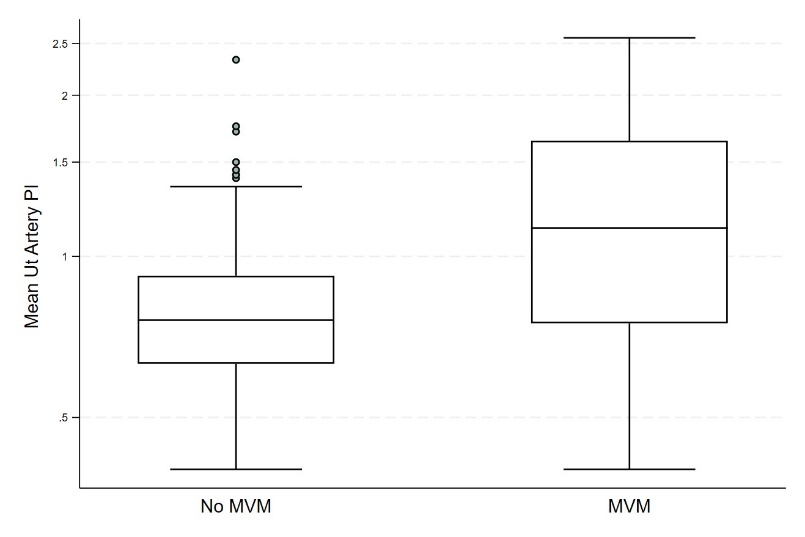 |
| 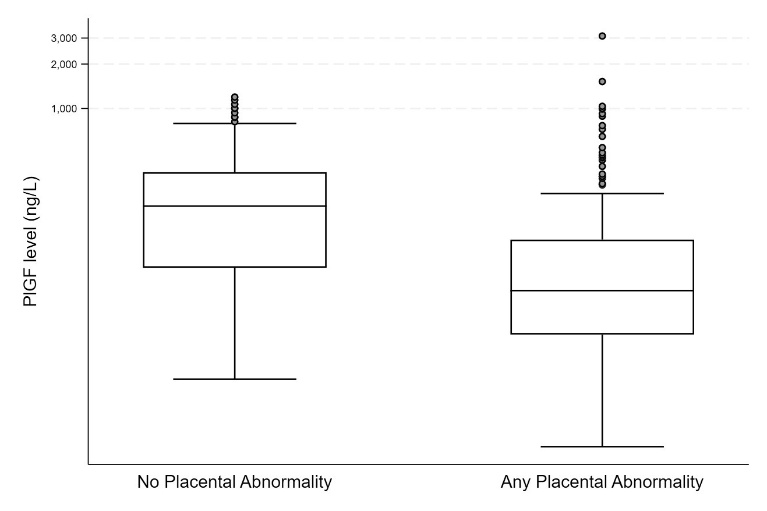 | 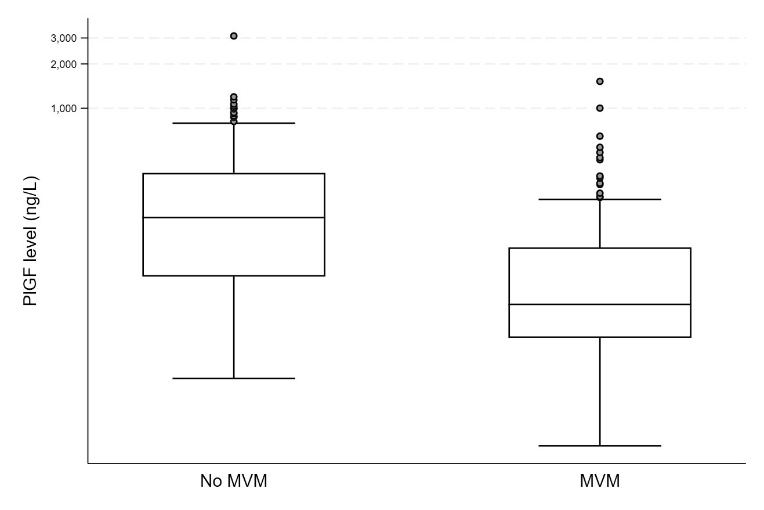 |
| 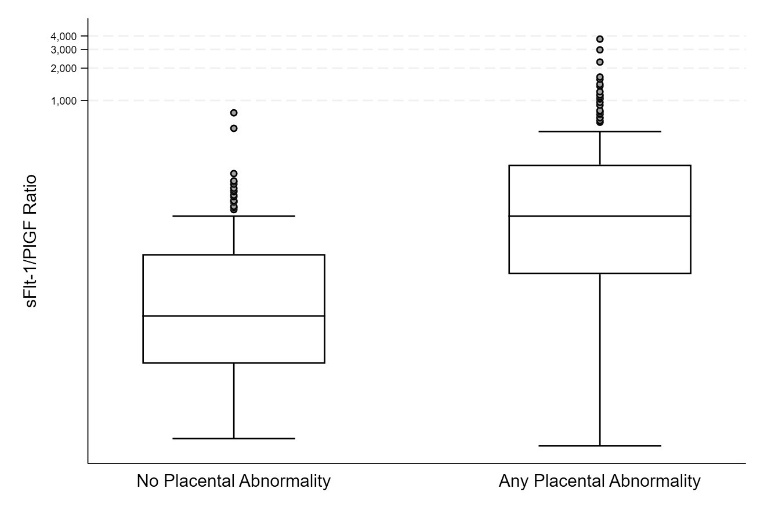 | 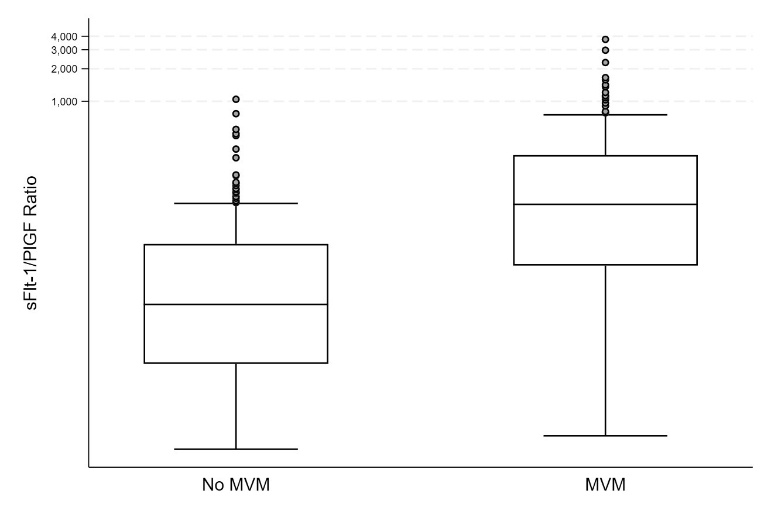 |

**Figure S1** Box-and-whiskers plots of raw values of fetoplacental Doppler parameters (cerebroplacental ratio (CPR); umbilical artery pulsatility index (PI); mean uterine artery PI) and placental biomarkers (placental growth factor (PlGF); and soluble fms-like tyrosine kinase-1 (sFlt-1)/PlGF ratio) at last measurement before birth in pregnancies with small-for-gestational age fetus, stratified by presence or absence of any placental abnormality and of maternal vascular malperfusion (MVM). Boxes show median and interquartile range (IQR), whiskers show 1.5 x IQR and circles are outliers.
